# Suppression of c-Myc and RRM2 expression in pancreatic cancer cells by the sphingosine kinase-2 inhibitor ABC294640

**DOI:** 10.18632/oncotarget.11112

**Published:** 2016-08-08

**Authors:** Clayton S. Lewis, Christina Voelkel-Johnson, Charles D. Smith

**Affiliations:** ^1^ Medical University of South Carolina, Department of Drug Discovery and Pharmaceutical Sciences, Charleston, South Carolina, USA; ^2^ Medical University of South Carolina, Department of Microbiology and Immunology, Charleston, South Carolina, USA; ^3^ Apogee Biotechnology Corporation, Hummelstown, Pennsylvania, USA

**Keywords:** sphingosine kinase-2, gemcitabine, pancreatic cancer, c-Myc, ribonucleotide reductase

## Abstract

Pancreatic cancer remains extremely difficult to treat, with the average lifespan following diagnosis being only 3-6 months, resulting in a death to incidence ratio of 0.94. A major reason for this high mortality rate is resistance to the main chemotherapeutic agent used to treat this disease, gemcitabine. Alterations in nucleoside and gemcitabine metabolism, specifically over-expression of ribonucleotide reductase, have been implicated as a major mechanism of resistance to this drug. Here, we show that inhibition of sphingosine kinase-2 by the specific inhibitor ABC294640 is synergistically cytotoxic with gemcitabine toward three human pancreatic cancer cell lines. Treatment with ABC294640 results in decreased expression of both *RRM2* and *MYC* in all three cell lines. Additionally, expression of c-Myc protein and phosphorylation of Rb at S780 both decrease in a dose-dependent manner in response to ABC294640, while acetylation of H3-K9 and p21 levels increase. Pretreatment with the protein phosphatase 1 inhibitor okadaic acid or the ceramide synthase inhibitor fumonisin B1 fails to prevent the effects of ABC294640 on Rb phosphorylation. These data indicate a role for sphingosine kinase-2 in E2F and c-Myc mediated transcription through alteration of histone acetylation and p21 expression. These effects of ABC294640 suggest that it may be an effective agent for pancreatic cancer, particularly in combination with gemcitabine.

## INTRODUCTION

For the last ten years the incidence rate of pancreatic cancer has been rising in the US while the five year survival rate remains near 6% [1]. The two main reasons for this alarming statistic are the lack of early warning signs of the disease which leads to a delay in diagnosis, often after the cancer has already grown beyond the borders of the pancreas, and resistance to the mainline chemotherapeutic drug, gemcitabine, a di-fluorinated nucleoside analog of deoxycytidine. After intracellular phosphorylation, gemcitabine induces cell death predominantly by incorporating into the DNA, leading to strand termination and subsequent apoptosis [2]. Chemoresistance to gemcitabine, whether inherent or acquired, occurs most often through modification of gemcitabine metabolism (reviewed by Bergman et al. [3]), most notably through overexpression of the catalytic subunit (RRM2) of ribonucleotide reductase.

Ribonucleotide reductase (RR) catalyzes the reduction of ribonucleotides yielding deoxyribonucleotides, and is the rate-limiting step in DNA synthesis. Its enzymatic activity is controlled mainly through transcriptional regulation [4]. A gemcitabine resistant cell line generated by incremental increases in exposure to gemcitabine was found to have a 9- and 2-fold increase in RRM2 mRNA and protein expression, respectively [5]. Additionally, expression levels of RRM2 in tumors were shown to be predictive of treatment responsiveness to gemcitabine [6]. The inhibition of RRM2 by RNAi technology [7] or exposure to flavopiridol [8], an upstream inhibitor of RRM2 transcription, has been shown to restore sensitivity to gemcitabine.

Another key protein that regulates pancreatic cancer cell sensitivity to gemcitabine is c-Myc, a central transcription factor with a plethora of target genes that play roles in proliferation, mitochondrial biogenesis, and glucose metabolism. The MYC proto-oncogene is overexpressed in many types of cancer [9], including pancreatic cancer [10] where it has been shown to decrease sensitivity to gemcitabine [11, 12].

Sphingolipids are important signaling lipids involved in several key cellular processes. The *de* *novo* synthesis of sphingolipids begins with the palmitoylation of serine yielding 3-ketodihydrosphingosine, which is reduced to dihydrosphingosine (dhSph), which is acylated to produce dihydroceramide (dhCer). dhCer is converted to ceramide (Cer) by dihydroceramide desaturase, which can then be further metabolized to additional types of sphingolipids. Of most importance to this research, however, is the ceramidase-mediated cleavage of ceramide to yield sphingosine. Sphingosine can be phosphorylated by one of two sphingosine kinases (SphK1 and SphK2) to form sphingosine-1-phosphate (S1P) which is degraded by S1P-lyase. Ceramide, sphingosine and S1P regulate reciprocal signaling events such that excess sphingosine and/or ceramide induce apoptosis in tumor cells, while S1P is pro-proliferation (reviewed by Hannun et al. [13]).

Sphingolipids have previously been shown to affect upstream modulators of both RRM2 and c-Myc transcription. Both MYC [14, 15] and RRM2 [16, 17] have E2F binding sites in their promoters subjecting them to partial control by the retinoblastoma family of proteins. Specifically, hypophosphorylated Rb binds E2Fs preventing the transcription of its target genes [18]. Two classes of proteins determine the phosphorylation status of Rb. Cyclin dependent kinases are responsible for phosphorylation[19] while protein phosphatases dephosphorylate Rb [20]. The addition of exogenous sphingosine induces the dephosphorylation of Rb, and addition of the ceramidase inhibitor fumonisin B1 potentiates this response [21]. Addition of C_2_-ceramide also leads to the dephosphorylation of Rb as a result of an increase in p21 expression and a corresponding decrease in the expression of MYC[15]. Therefore, modulation of sphingolipid signaling may provide a novel means of sensitizing pancreatic tumor cells to gemcitabine.

We have previously shown that the sphingosine kinase 2 selective inhibitor ABC294640 has broad anti-tumor activity [22, 23], that its effects mimic SphK2 ablation, and that loss of SphK2 impacts tumor growth more profoundly than loss of SphK1 [24]. Additionally, ABC294640 has been shown to decrease intracellular c-Myc levels in a variety of cancer cell types [25, 26]. Here, we show ABC294640 increases gemcitabine sensitivity in three human pancreatic cancer cell lines (BxPC-3, MiaPaCa-2, and Panc-1), and that this is associated with increased acetylation of lysine 9 on histone 3 and increases in p21. This reduces the phosphorylation of Rb, leading to the sequestration of E2F1 and thereby decreasing the expression of both MYC and RRM2. These data provide mechanistic rationale for combination of ABC294640 with gemcitabine as a new therapeutic approach to pancreatic cancer.

## RESULTS

### ABC294640 enhances the cytotoxicity of gemcitabine toward pancreatic cancer cells

**Figure 1:**
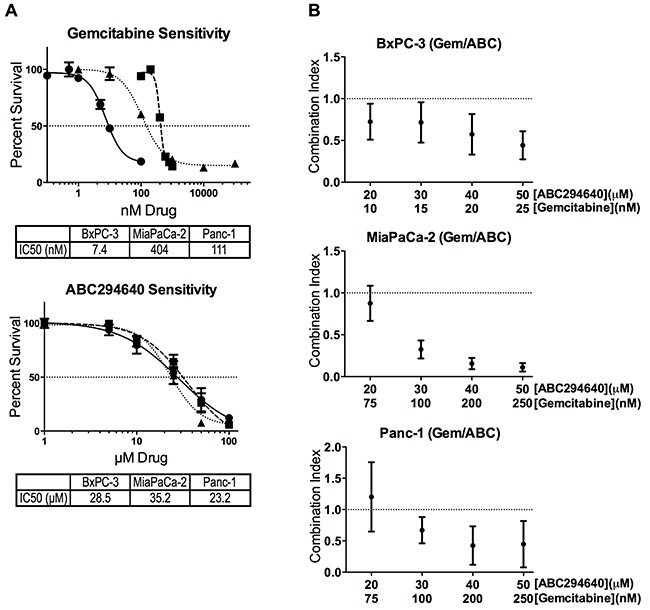
Cytotoxicity of ABC294640 and gemcitabine alone and in combination **Panel A.** BxPC3 (●), MiaPaCa-2 (■) or Panc-1 (▲) pancreatic cell lines were exposed to gemcitabine and/or ABC294640 at the indicated concentrations for 96-hours. Cell survival was then quantified using the SRB assay. **Panel B.** Combination index values of the combination of ABC294640 and gemcitabine in three pancreatic cell lines were calculated using Calcusyn. All experiments were repeated three times, and values represent mean ± SEM.

We have previously described the anticancer effects of multiple SphK inhibitors [27], and ABC294640, an SphK2 selective inhibitor, was found to have broad anti-cancer activity [22]. Its combination with the established anticancer drug sorafenib results in synergistic cytotoxicity toward A498 cells, as well as the pancreatic adenocarcinoma cell line, BxPC-3 [28]. We have now combined ABC294640 with the nucleoside analog gemcitabine to evaluate their usefulness together as a potential therapy for pancreatic cancer. We first established the 96-hour IC_50_ concentrations for gemcitabine and ABC294640 (single-agent) in BxPC-3, MiaPaCa-2 and Panc-1 cells as 7.4, 404 and 111 nM for gemcitabine, and 28.5, 35.2 and 23.2 μM for ABC294640, respectively (Figure 1a). Thus, the pancreatic cancer cell lines have a much greater range of sensitivity to gemcitabine than they do to ABC294640. Additionally, each cell line was treated with multiple concentrations of ABC294640 and gemcitabine and cell survival was quantified after 96 hours. Combination of ABC294640 with gemcitabine resulted in synergistic cell killing (Combination Index <1.0) in all three cell lines at and above the IC_50_ for each individual drug (Figure 1b). Interestingly, combining ABC294640 and gemcitabine in MiaPaCa-2 cells, the cell line with the highest intrinsic resistance to gemcitabine, resulted in the lowest CI values, which are indicative of strong synergism.

### ABC294640 suppresses the expression of c-Myc and RRM2 in pancreatic cancer cells

**Figure 2:**
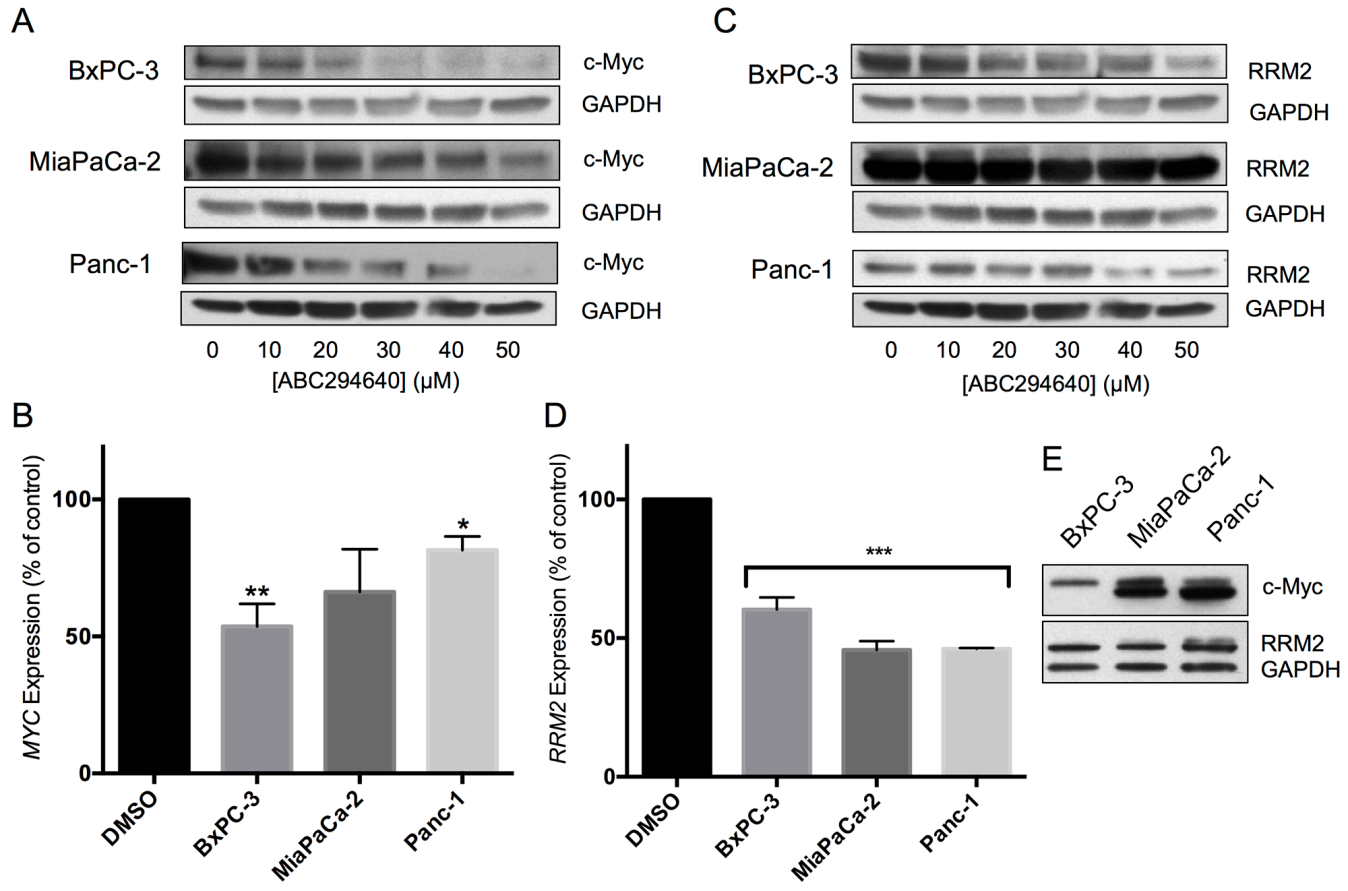
Effects of ABC294640 on expression of c-Myc and RRM2 **Panels A** and **C.** BxPC-3, MiaPaCa-2 and Panc-1 cells were treated with the indicated concentration of ABC294640 for 24 hr. Cells were then harvested and analyzed for protein expression levels of c-Myc and GAPDH (Panel A) or RRM2 and GAPDH (Panel C). Western blots are representative of three independent experiments. **Panels B** and **D.** BxPC-3, MiaPaCa-2 and Panc-1 cells were treated with 30 μM ABC294640 for 24 hr. Cells were then harvested and analyzed for mRNA expression levels of *MYC* (Panel B) and *RRM2* (Panel D) (p < 0.05*,0.005**, or 0.0001***). qRT-PCR was conducted in two independent experiments. **Panel E.** Untreated BxPC-3, MiaPaCa-2 and Panc-1 cell lysates were analyzed for expression of c-Myc by western blotting.

Recently, it was shown that inhibition of SphK2 by ABC294640 results in decreased c-Myc expression in acute lymphoblastic leukemia [25], multiple myeloma [26] and prostate cancer [29, 30] cells. Here, we show that ABC294640 causes a concentration-dependent decrease in *MYC* mRNA, as well as c-Myc protein, in three pancreatic cell lines. BxPC-3 cells were most responsive, experiencing a near 50% reduction in mRNA and protein expression when treated with 30 μM ABC294640 (Figure 2a & 2b). MiaPaCa-2 and Panc-1 cells had more moderate decreases in mRNA levels, but demonstrated similar decreases in protein expression when treated with 30 μM ABC294640. When comparing the native protein expression levels of c-Myc among the three cell lines, untreated BxPC-3 cells were found to express substantially less c-Myc than the other two cell lines (Figure 2e).

Overexpression of the catalytic subunit of ribonucleotide reductase (RRM2) has often been cited as one of the major mechanisms for gemcitabine resistance [5–7]. Therefore, we examined the effect of ABC294640 on *RRM2* transcription and translation in the pancreatic cell line panel. Treatment of the BxPc-3, MiaPaCa-2 or Panc-1 cells with ABC294640 resulted in a near 50% reduction in *RRM2* mRNA in all three cell lines, which was matched by decreases in RRM2 protein expression (Figure 2c & 2d). Therefore, ABC294640 causes dose-dependent suppression of two key genes, *MYC* and *RMM2*, that drive pancreatic cancer growth and resistance to chemotherapy.

**Figure 3:**
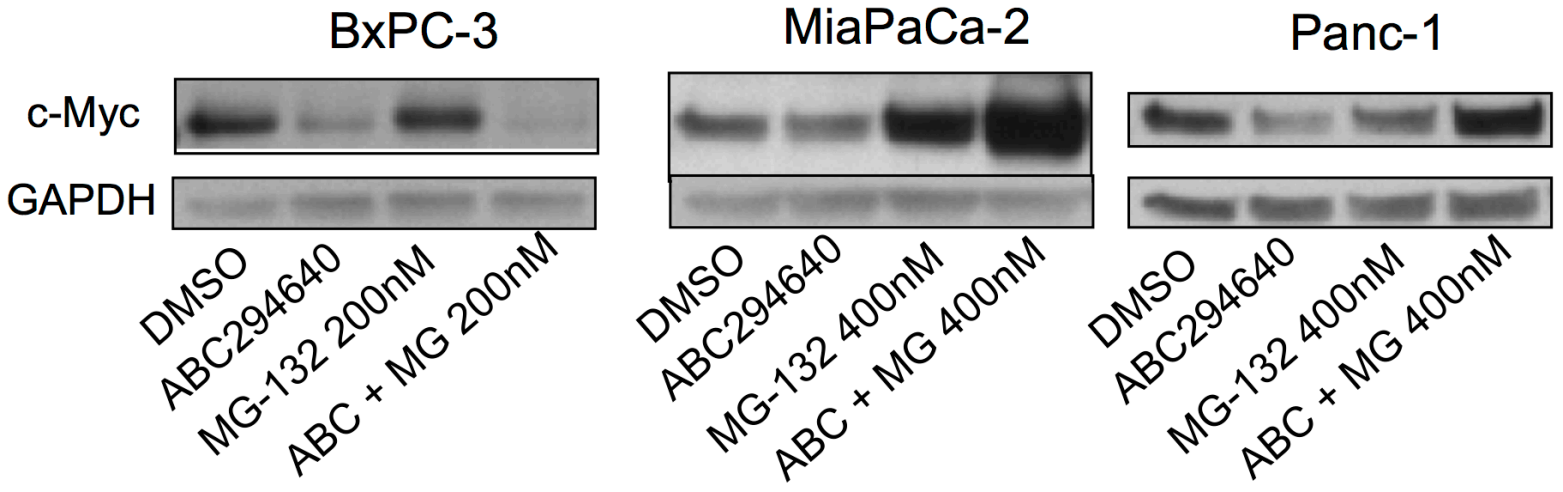
Effects of ABC294640 on proteasomal degradation of c-Myc The indicated pancreatic cancer cells were incubated with DMSO (control) or MG-132 for 2 hr and then treated for 24 hr with 30 μM (BxPC-3) or 40 μM (MiaPaCa-2 and Panc-1) ABC294640. Cells were then harvested and analyzed for protein expression levels of c-Myc and GAPDH. Western blots are representative of two independent experiments.

Because the role of proteasomal degradation in the suppression of c-Myc expression appears to vary among cell types [25, 26, 29], proteasomal degradation was addressed as a potential mechanism for c-Myc suppression in the panel of pancreatic cancer cell lines. Cells were treated with the respective IC_50_ dose of ABC294640 with and without the proteasome inhibitor MG-132. As indicated in Figure 3, MG-132 did not abrogate the effect of ABC294640 in BxPC-3 cells, indicting a proteasome-independent pathway for c-Myc reduction in these cells. However, MG-132 substantially increased the accumulation of c-Myc in both MiaPaCa-2 and Panc-1 cells following ABC294640 treatment (Figure 3), suggesting that ABC294640-mediated reduction of c-Myc in these cells is at least partially by enhanced proteolysis.

### ABC294640-regulation of c-Myc is mediated by suppression of Rb phosphorylation

**Figure 4:**
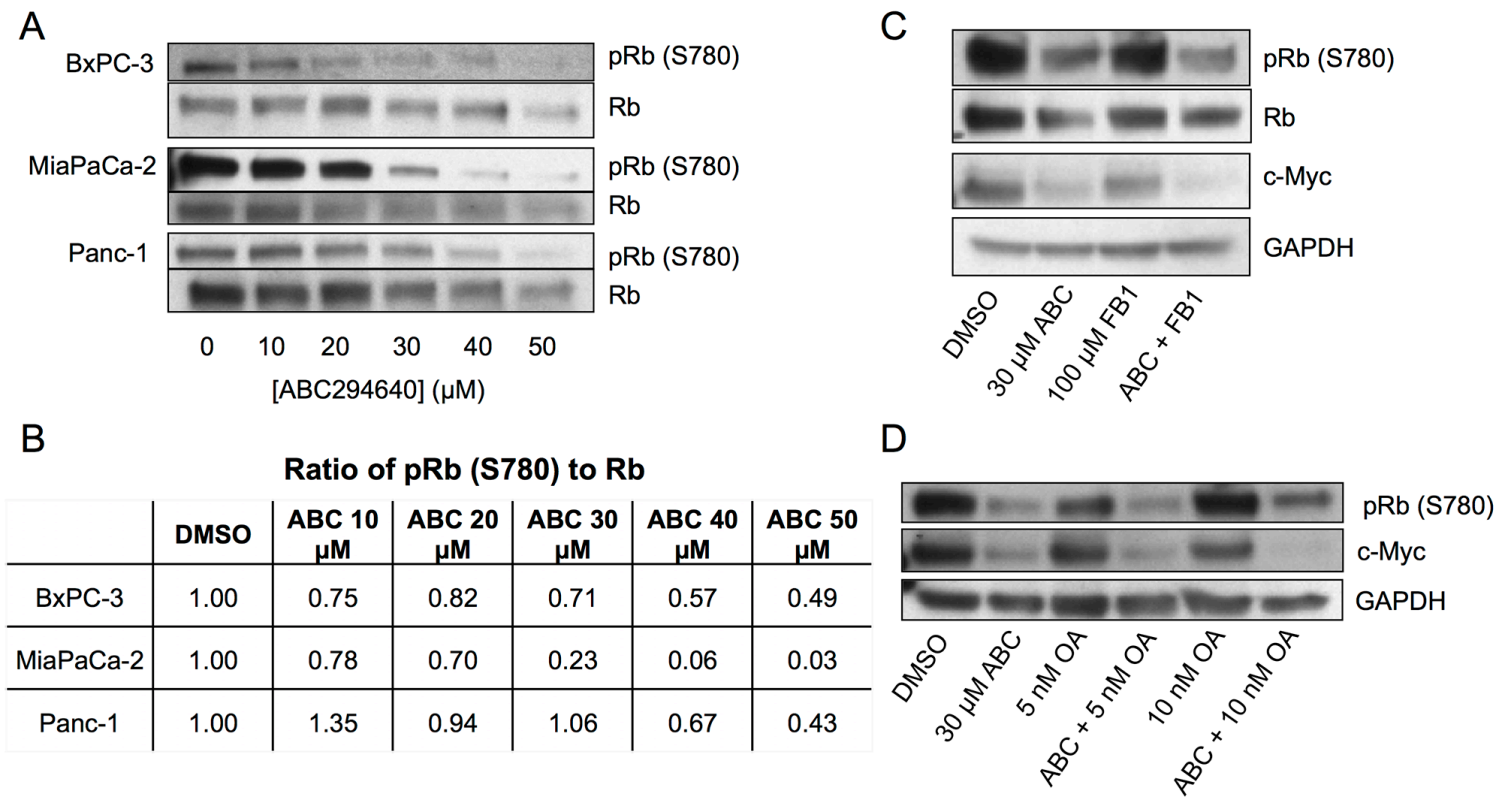
Effects of ABC294640 on phosphorylation of Rb **Panel A.** BxPC-3, MiaPaCa-2 and Panc-1 cells were treated with the indicated concentration of ABC294640 for 24 hr. Cells were then harvested and analyzed for protein expression levels of total Rb and pRb (S780) by western blotting. **Panel B.** The ratio of pRb S780 to Rb in BxPC-3, MiaPaCa-2, and Panc-1 following treatment with the indicated concentration of ABC294640 for 24 hr. **Panel C.** BxPC-3 cells were treated with 100 μM fumonisin B1 and/or 30 μM ABC294640 for 24 hr. Cells were then harvested and analyzed for protein expression levels of total Rb, pRb (S780), c-Myc and GAPDH by western blotting. **Panel D.** BxPC-3 cells were treated with the indicated concentration of OA and/or 30 μM ABC294640 for 24 hr. Cells were then harvested and analyzed for protein expression levels of pRb (S780), c-Myc and GPDH by western blotting. Western blots are representative of three independent experiments, the ratios in Panel B are derived from the blots in Panel A.

The Retinoblastoma protein, Rb, is a tumor suppressor controlling the cell cycle by sequestering several E2F family transcription factors (E2F1-5, hereafter termed E2F). Previous studies have shown that E2F can control the transcription of c-Myc [15, 31] and RRM2 [14, 16]. Phosphorylation of multiple serine residues of Rb causes release of E2F, and is correlated with the activation state of E2F [18]. Importantly, sphingosine, but not S1P has also been shown to activate Rb [21]. Because of this linkage with sphingolipid metabolism, we examined the effects of ABC294640 on the phosphorylation state of Rb as an indicator of E2F activity. As indicated in Figure 4a and 4b, treatment of BxPC-3, MiaPaCa-2 or Panc-1 cells with increasing concentrations of ABC294640 resulted in dose-dependent decreases in the phosphorylation of Rb at S780, which would result in increased sequestration and inhibition of E2F. Interestingly, MiaPaCa-2 cells (which are the most resistant to gemcitabine (Figure 1a)) demonstrated the most extensive suppression of Rb phosphorylation by ABC294640 among the pancreatic cancer cell lines. Therefore, lack of sensitivity to gemcitabine does not prevent the signaling effects from inhibition of SphK2.

It has previously been shown that exogenous short-chain ceramides cause accumulation of long-chain ceramides via the salvage pathway, leading to inhibition of c-Myc in A549 cells [32]. Ceramides have been shown to directly activate protein phosphatase 1 (PP-1) which dephosphorylates Rb both *in vitro* [33] and *in vivo* [34]. Similarly, previous studies showed that exposing cells to sphingosine caused a reduction in Rb phosphorylation [21]. Combining these findings suggests that elevation of ceramide and/or sphingosine levels may activate PP-1, causing dephosphorylation of Rb, leading to the sequestration of E2F and decreased transcription of c-Myc. Because ABC294640 inhibits conversion of sphingosine to S1P, we sought to determine whether elevated sphingosine is responsible for the observed decrease in pRb (S780) in ABC294640-treated cells, or whether the increased sphingosine is salvaged back to ceramide (by ceramide synthases) which may activate protein phosphatases. Therefore, BxPC-3 cells were treated with ABC294640 and fumonisin B1 (FB1), an inhibitor of ceramide synthases, and examined for changes in the phosphorylation status of Rb as well as changes in c-Myc. As indicated in Figure 4c, FB1 did not prevent the dephosphorylation of Rb or the downstream suppression of c-Myc by ABC294640. Based on these data and those of Pushkareva et al regarding the relationship between sphingosine, ceramide, ceramidase and Rb phosphorylation [21], it is reasonable to conclude that ABC294640 reduction of Rb phosphorylation is mediated by sphingosine rather than a ceramide-dependent mechanism.

To further investigate the mechanism for the decrease in Rb phosphorylation, we inhibited PP-1 in BxPC-3 cells using the established phosphatase inhibitor okadaic acid (OA) [34–36]. As indicated in Figure 4d, OA alone caused a slight increase in Rb phosphorylation at S780, but did not substantially attenuate the dephosphorylation of Rb caused by ABC294640. Furthermore, OA did not prevent the reduction of c-Myc expression in cells also treated with ABC294640. These data indicate that the decrease in Rb phosphorylation and c-Myc expression by ABC294640 is not due to increased protein phosphatase activity.

### ABC294640 induces p21 expression and histone H3 acetylation in pancreatic cancer cells

**Figure 5:**
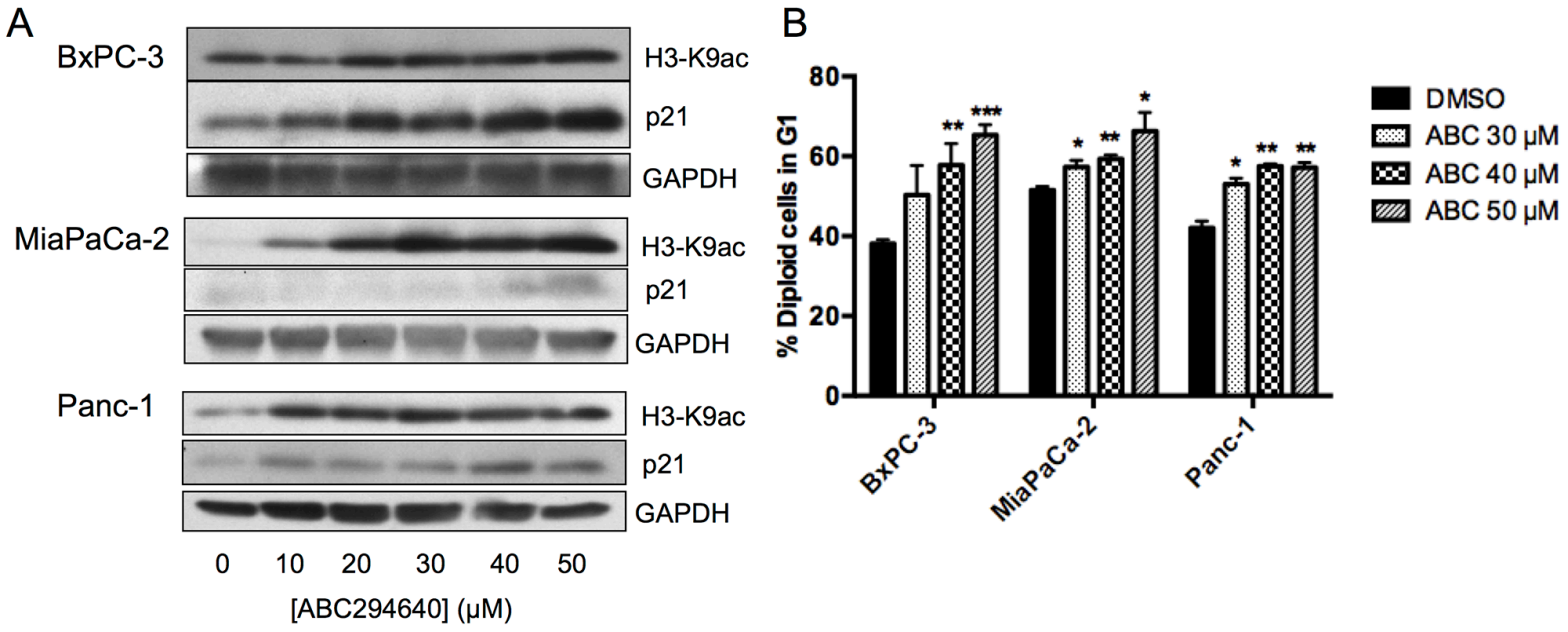
Effects of ABC294640 on H3K9ac, p21, and cell cycle **Panel A.** BxPC-3, MiaPaCa-2 and Panc-1 cells were treated with the indicated concentration of ABC294640 for 24 hr. Cells were then harvested and analyzed for protein expression levels of H3-K9ac, p21 and GAPDH by western blotting. Western blots shown are representative of three independent experiments. **Panel B.** BxPC-3, MiaPaCa-2 and Panc-1 cells were treated with the indicated concentration of ABC294640 for 24 hr. Cells were then harvested and analyzed by flow cytometry, and the percentage of cells in G1 is indicated (p < 0.05*,0.005**, or 0.0005***). These are the combined results of three independent experiments.

Rb activity is regulated by its phosphorylation state, which is maintained by a balance between cyclin-dependent kinases (cdks) and protein phosphatases 1 and 2. Because OA failed to prevent the hypophosphorylation of Rb in response to ABC294640, this response may be mediated by inhibition of cdks. Therefore, the effects of ABC294640 on the cdk inhibitor p21, which binds to the cyclinD/cdk4/6 complex inhibiting the kinase activity, were assessed. Treatment of BxPC-3, MiaPaCa-2 or Panc-1 cells with increasing concentrations of ABC294640 resulted in concentration-dependent increases in p21 expression as determined by immunoblotting (Figure 5a). Because elevation of p21 is associated with G1 arrest, we examined the effect of ABC294640 on the cell cycle distributions of the three pancreatic cancer cell lines. As indicated in Figure 5b, increasing concentrations of ABC294640 resulted in increasing numbers of cells in the G1 phase for all three cell lines. Elevation of p21 is often the result of increased p53 activity, and all three of the pancreatic cancer cell lines under study have inactivating mutations in p53. Nonetheless, we examined the impact of ABC294640 on total p53 and phospho-p53 (S15) levels, and found no observable changes in either after ABC294640 treatment (data not shown).

Increasing lysine acetylation by inhibition of histone deacetylases (HDACs) has been shown to increase the expression of p21 [37] and to decrease the expression of c-Myc [38–42]. Because both of these changes occur in cells treated with ABC294640, we assessed the effects of the drug on levels of lysine-9 acetylation of histone H3 (H3-K9ac) in each of the pancreatic cancer cell lines. Treatment with ABC294640 caused dose-dependent elevations of H3-K9ac, particularly in MiaPaCa-2and Panc-1 cells (Figure 5a). These data are consistent with the model in which ABC294640 depletes the nuclear pool of S1P, thereby suppressing HDAC activity and altering protein expression.

## DISCUSSION

Acquired resistance to gemcitabine is a major factor in the poor response of pancreatic tumors to current chemotherapy protocols. Here, we show that the inhibition of SphK2 by ABC294640 synergizes with gemcitabine to increase pancreatic cancer cell killing. Interestingly, this synergy was most pronounced in the most resistant cell line, MiaPaCa-2, where synergy was observed well below the IC_50_ concentration for gemcitabine. The present studies provide the first evidence that inhibition of SphK2 by ABC294640 directly affects the metabolism of gemcitabine through the down-regulation of RMM2 expression. Ribonucleotide reductase overexpression results in an increased pool of dNTPs that outcompete gemcitabine for incorporation into elongating DNA during replication. Therefore, overexpression of RRM2 is a major mechanism for gemcitabine resistance [5–7]. The synergistic killing of pancreatic tumor cells by combined treatment with ABC294640 plus gemcitabine is likely mediated by suppression of RRM2-mediated resistance in parallel with suppression of proliferation by the SphK2 inhibitor.

Improved cell killing by combining ABC294640 with gemcitabine is consistent with our previous findings and those of others that demonstrate that ABC294640 can be effectively combined with other cancer drugs. For example, Antoon et al. demonstrated the ability of ABC294640 to overcome NF-κB-mediated chemoresistance in breast cancer [43]. This is highly relevant to pancreatic cancer because NF-κB is overexpressed in 70% of patients [44] and is thought to be an additonal mechanism for gemcitabine resistance [45]. Additionally, treatment of ovarian cancer cells with ABC294640 caused increased expression of BAX [46], thereby tilting the balance of the bcl-2 family of proteins, which are known to contribute to gemcitabine resistance [47], in the pro-apoptotic direction. The accumulated data provide a compelling argument for combination of ABC294640 and gemcitabine for the treatment of pancreatic cancer.

c-Myc is a transcription factor downstream of p38 kinase with well-documented oncogenic activity (reviewed by Miller et al[9]). Overexpression of c-Myc induces the transcription of genes that promote proliferation, stimulate mitochondrial biogenesis, and regulate glucose metabolism. Because direct targeting of c-Myc with new drugs has not yet been successful, reducing its expression and activity by targeting upstream regulators may be the most practical current approach to ablating the tumorigenic effects of c-Myc. Consistent with recent publications in other cell types [25, 26, 29, 30], we demonstrate herein that ABC294640 down-regulates the expression of c-Myc in pancreatic cancer cells. Overexpression of c-Myc in the pancreas was shown to produce ductal adenocarcinoma in transgenic mice [48], and cells over-expressing c-Myc have been reported to be resistant to gemcitabine [12]. ABC294640-mediated reduction of c-Myc by enhanced proteasomal degradation has been observed in some cell lines [26]. Our data show that reduction of c-Myc by ABC294640 in BxPC-3 cells is not affected by proteasome inhibition; whereas in MiaPaCa-2 and Panc-1 cells, addition of ABC294640 following pretreatment with MG-132 increased levels of c-Myc. This paradoxical increase in c-Myc expression does not overcome the suppression of proliferation by ABC294640, and may reflect the cells' attempt to compensate for the antiproliferative effects of the drug. In addition to effects on proliferation, suppression of c-Myc may directly reduce RMM2 expression since the *RMM2* promoter contains a stimulatory c-Myc binding motif [49].

Herein, we show that inhibition of SphK2 by ABC294640 results in an increase in p21 expression that correlates with G1 arrest in three human pancreatic cancer cell lines. This agrees with our earlier findings which showed that SphK2 inhibition via siRNA increases in the percentage of A498 cells in the G1 phase [24]. This is also consistent with studies with Hs 27 human fibroblasts that demonstrated that addition of exogenous C_2_-ceramide results in increases in p21 and growth arrest [15]. However, the fate of p21 expression as a result of SphK2 inhibition may be cell type specific since it is reported that SphK2 siRNA decreases p21 expression in MCF7 breast cancer cells [50]. c-Myc also acts as a transcriptional suppressor of p21 [51], and so downregulation of c-Myc by ABC294640 leads to a release of p21 suppression. Suppression of p21 expression by ABC294640 is predictably linked to reduction in the phosphorylation status of Rb, and a consequent suppression of E2F transcriptional activity. Additionally, the E2F transcription factors are transcriptional targets of c-Myc [52]; and therefore, Myc suppression can also indirectly decrease the transcription of E2F target genes. Given the roles of genes regulated by E2F, attenuation of its activities is an attractive approach for chemotherapy. Most directly relevant to the present study is the observation that transcription of RRM2 is partially dependent on E2F transcription factor activity [14, 16], mediated by two E2F binding sites in the *RRM2* promoter [49].

Data presented herein indicate a key role for suppression of HDAC activity by ABC294640, and this is consistent with previous studies indicating that HDAC inhibitors increase the efficacy of gemcitabine [53]. Additionally, the observed effects of ABC294640 on increasing p21 [37] and diminished c-Myc are indicative of HDAC inhibition [38–42, 54]. Although SphK2 and SphK2-derived S1P have been previously suggested to inhibit HDAC activity [50], as demonstrated in Figure 5, ABC294640 increases H3-K9ac which indicates inhibition of HDAC activity. We postulate that inhibition of SphK2 by ABC294640 reduces the association of nuclear S1P with HDAC2 leading to a decrease in the binding of H3-K9ac to the *MYC* promoter and decreased c-Myc expression [25]. Therefore, ABC294640 inhibits *MYC* transcription on two fronts, ie HDAC inhibition directly decreases *MYC* transcription and E2F-regulated transcription is also suppressed.

**Figure 6:**
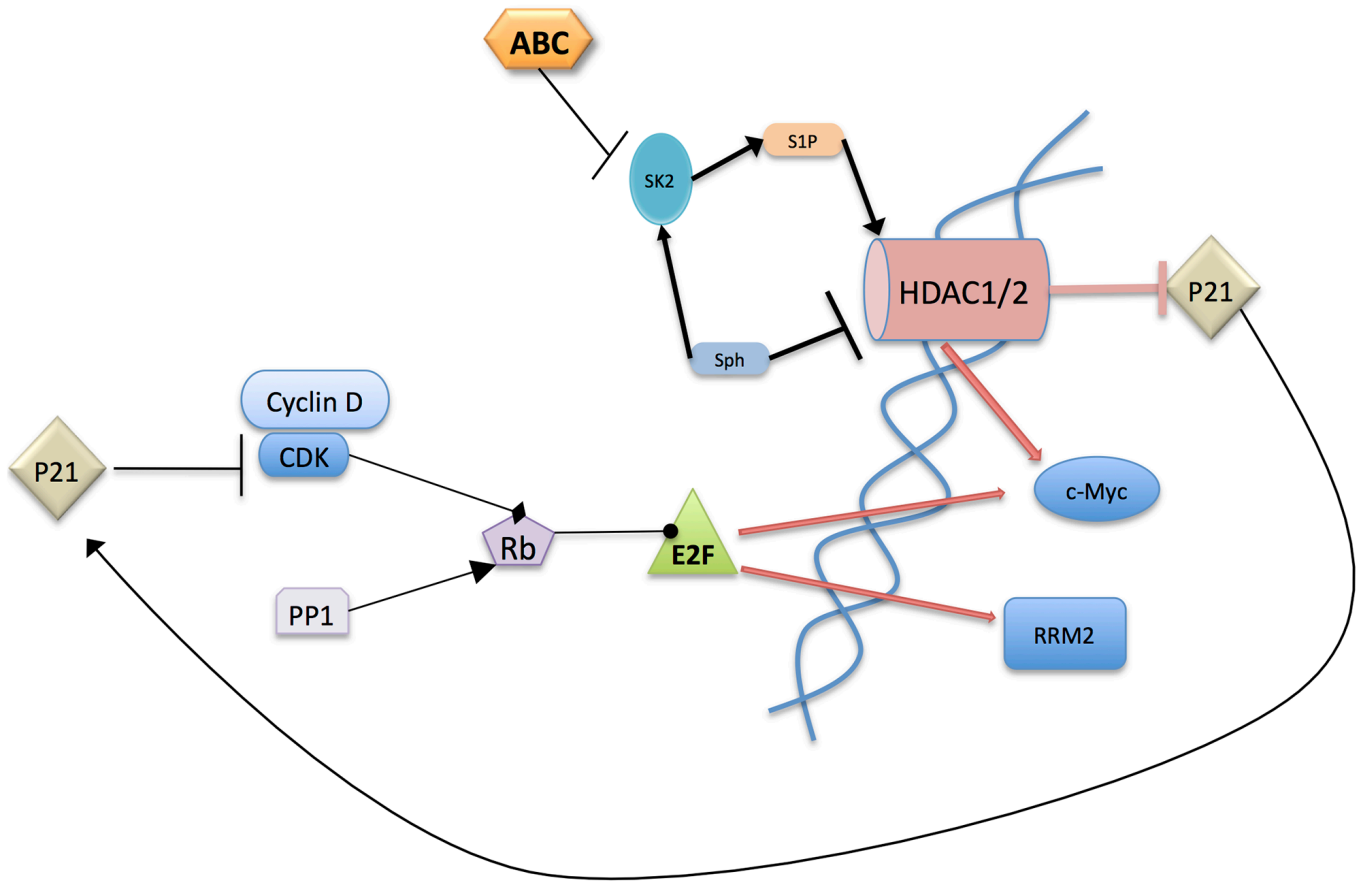
Postulated mechanism of action of ABC294640 ABC294640 acts by inhibiting SphK2 in the nucleus leading to HDAC inhibition because of loss of nuclear S1P and/or elevation of nuclear sphingosine levels. This results in an increase in p21 which binds CDK/Cyclin-D1 complexes preventing the phosphorylation of Rb. The resulting hypophosphorylated Rb binds E2F preventing its transcriptional activity. It should also be noted that c-Myc inhibits the transcription of p21 and enhances the transcription of CDKs, Cyclin-D1, and E2F1, 2 and 3. The overall result is a decrease in proliferative signaling thereby resulting in inhibition of cancer growth.

In total, data presented herein further underscore the importance of sphingolipid metabolism in regulating both cell proliferation and pancreatic tumor cell sensitivity to gemcitabine. Specifically, these studies provide a new model in which nuclear S1P derived from SphK2 promotes the expression of the key pro-proliferative and metabolism genes *MYC* and *RRM2* (Figure 6). A central role for inhibition of HDAC activity by ABC294640 resulting in multifaceted, but complimentary, downstream modulation of c-Myc, p21, phospho-Rb and RRM2 expression all combine to attenuate tumor cell proliferation and increase sensitivity to gemcitabine. Specifically, the mechanism by which ABC294640 suppresses c-Myc and RRM2 expression appears to be at least partially mediated by inhibition of HDAC activity, which leads to an increase in p21 transcription and decrease in *MYC* transcription. The increase in p21 results in a decrease in Rb phosphorylation due to the inhibition of cyclin/CDK complexes. Hypophosphorylated Rb sequesters E2F preventing its transcriptional activity, resulting in a decrease in c-Myc and RRM2. c-Myc has transcriptional impact on multiple members of this signaling pathway, creating an amplification process and thereby ensuring cancer cell death.

As a whole, these data contribute to our understanding of the roles of SphK2 in tumor biology, further supporting critical roles for sphingolipids as regulators of gene expression. We have also elucidated a mechanism through which synergy between ABC294640 and gemcitabine may occur. Because, few drugs counteract multiple modes of drug resistance, we believe the accumulated information supports the future combined use of gemcitabine and ABC294640 for pancreatic cancer chemotherapy.

## MATERIALS AND METHODS

### Cell lines

BxPC-3, MiaPaCa-2 and Panc-1 cells were purchased from the American Type Culture Collection, and were grown at 37°C in a humidified atmosphere containing 5% CO_2_. BxPC-3 cells were grown in RPMI 1640 medium containing 10% fetal bovine serum (FBS) and 25 μg/mL gentamicin. MiaPaCa-2 cells were grown in DMEM containing 10% FBS, 5% horse serum and 25 μg/mL gentamicin. Panc-1 cells were grown in DMEM containing 10% FBS and 25 μg/mL gentamicin.

### Reagents

ABC294640 (cGMP grade) was provided by Apogee Biotechnology Corporation. Gemcitabine HCl was purchased from Sigma-Aldrich. The following antibodies were purchased from: Cell Signaling - c-Myc (9402s), p21 (2947P), pRb (S780) (9307P), Rb (9309P), GAPDH (2118s), Santa Cruz - Ubiquitin (sc-8017) and Abcam - RRM2 (ab57653). The following primers were purchased from Qiagen: MYC (PPH00100B), RRM2 (PPH14649A), SPHK1 (PPH02491A), and SPHK2 (PPH21192A) GAPDH(PPH00150F).

### Cytotoxicity assays

96-well plates were seeded with 3,000 cells/well and incubated overnight. Cells were then treated with varying concentrations of gemcitabine and/or ABC294640 and after 96 hours of treatment, the medium was removed and the cells were washed with Phosphate-Buffered Saline (PBS) and fixed in 10% trichloroacetic acid (TCA) overnight at 4°C. The TCA was then removed and the cells were washed with water three times before adding sulpharhodamine B (4 g/L in 1% acetic acid) for 30 minutes. The cells were then de-stained with 1% acetic acid and left to dry. 100 μL of 10 mM TRIS was then added to each well and the absorbance at 560 nm was measured using a SpectraMax M5 platereader (Molecular Devices).

### Protein isolation and immunoblots

After 24 hours of treatment, cells grown in either 100 or 150 mm plates were washed three times with PBS and harvested into lysis buffer (50 mM Tris, 150 mM NaCl, 5 mM EDTA, 5 mM EGTA, 1% NP-40, pH=7.4 plus protease and phosphatase inhibitors) on ice. The lysates were then centrifuged at 23,000 x g for 25 minutes at 4°C, and each supernatant was transferred to another microcentrifuge tube. A BCA kit (Pierce) was used to determine protein concentration, and samples were then equalized using lysis buffer. For SDS-PAGE, samples containing 30 μg of protein were separated on 10-well 10% Mini-PROTEAN® TGX™ pre-cast gels (Bio-Rad) and then transferred to polyvinyl difluoride membranes using the Trans-Blot® SD Semi-Dry Electrophoretic Transfer Cell (Bio-Rad). The membranes were blocked for 1 hour in 5% bovine serum albumin (BSA) and then incubated with primary antibody for 1-2 hours according to the manufacturer's recommended dilution (typically 1:1000). After washing, the membrane was incubated with a horseradish peroxidase conjugated secondary antibody for 1 hour (1:30,000). After washing, 1 mL of an enhanced chemiluminescent substrate (Thermo Scientific) was applied to the membrane and imaging was carried out in a dark room with HyBlot® CL autoradiography film (Denville Scientific).

### Quantitative PCR

Following treatment, cells were harvested and total RNA was collected using the RNeasy kit (Qiagen) and quantified using a NanoDrop 1000 spectrophotometer (Thermo Scientific). RNA (1 μg) was used to synthesize cDNA using the SuperScript III First-Strand Synthesis System for RT-PCR (Invitrogen). The cDNA samples were aliquoted and combined with primers, nuclease-free water and SsoFast EvaGreen Supermix, and qRT-PCR was carried out on a MyiQ Real-Time PCR system (Bio-Rad). The cycling parameters consisted of 1 enzyme activating cycle of 95°C for 5 minutes, followed by 45 cycles of denaturation (95°C for 45 sec) and annealing/extension (60°C for 1 min). The cycle threshold was determined for each sample and the values were normalized to GAPDH.

### Statistics and graphing

All graphs, as well as IC_50_ calculations and treatment comparison statistics, were made using GraphPad Prism (version 5). Combination Index (CI) calculations were made using Calcusyn (Biosoft) following the software's designed protocol for synergy calculations.
